# Bacillus Calmette-Guerin therapy after the second transurethral resection significantly decreases recurrence in patients with new onset high-grade T1 bladder cancer

**DOI:** 10.1186/s12894-016-0126-x

**Published:** 2016-02-27

**Authors:** Keitaro Iida, Taku Naiki, Noriyasu Kawai, Toshiki Etani, Ryosuke Ando, Yosuke Ikegami, Takehiko Okamura, Hiroki Kubota, Atsushi Okada, Kenjiro Kohri, Takahiro Yasui

**Affiliations:** Department of Nephro-Urology, Nagoya City University, Graduate School of Medical Sciences, Kawasumi 1, Mizuho-cho, Mizuho-ku, 467-8601 Nagoya, Japan; Department of Urology, Daido Hospital, Aichi, Japan; Department of Urology, Nagoya City East Medical Center, Aichi, Japan; Department of Urology, Anjo Kosei Hospital, Aichi, Japan; Department of Urology, Kainan Hospital, Aichi, Japan

**Keywords:** BCG induction instillation, Intravesical recurrence, New onset, Second transurethral resection of bladder tumor, Urothelial carcinoma high grade

## Abstract

**Background:**

The purpose of this study was to assess the efficacy of Bacillus Calmette-Guerin (BCG) therapy after a second transurethral resection (TUR) in new onset high-grade T1 bladder cancer.

**Methods:**

From January 2008 to September 2013, 207 patients with new onset high-grade T1 bladder cancer after an initial TUR were treated at our university and at affiliated hospitals. Residual cancer rate, intravesical recurrence-free survival (RFS), and risk factors for intravesical recurrence were analyzed.

**Results:**

Among a total of 207 patients, 42 patients were treated with BCG therapy following a second TUR (group 1), 23 were treated with second TUR alone (group 2), 72 were treated with BCG alone (group 3), and 70 were treated without a second TUR or BCG. The median patients’ age was 72.0 years, and the median follow-up period was 33.5 months. The second TUR revealed that 34 patients (52 %) had residual cancer. Between groups 1 and 2 and groups 1 and 3, the differences in RFS were statistically significant (*p* = 0.002 and 0.045, respectively). In addition, BCG therapy was the most significant factor to predict RFS after the second TUR. Among the 31 patients whose pathology of the second TUR was pT0, only 1 of 12 patients (8 %) in group 1 and 11 of 19 patients (58 %) in group 2 had a recurrence.

**Conclusions:**

BCG instillation following a second TUR decreases intravesical recurrence, even if the pathology of the second TUR is pT0.

## Background

Among the non-muscle-invasive bladder cancers (NMIBCs), high-grade T1 bladder cancer presents high risk of intravesical recurrence after an initial transurethral resection (TUR). Bacillus Calmette-Guerin (BCG) induction instillation after an initial TUR is effective, reducing the risk of recurrence and progression [1–6]. A second TUR, which is defined as a repeated TUR performed within 2–8 weeks following an initial TUR, has been recommended since the 2000s for both resecting residual tumor and detecting staging error [1–6]. According to clinical guidelines, BCG induction instillations after a second TUR are recommended especially in cases with residual tumors in the second TUR [7]. A second TUR reduces the number of residual tumors and enhances the effects of intravesical BCG therapy; however, we are lacking large cohorts on the efficacy of BCG therapy when the pathology of the second TUR is pT0.

In this study, we performed an analysis of 207 patients with new onset high-grade T1 bladder cancer and evaluated the efficacy of BCG induction instillations following a second TUR.

## Methods

**Fig. 1:**
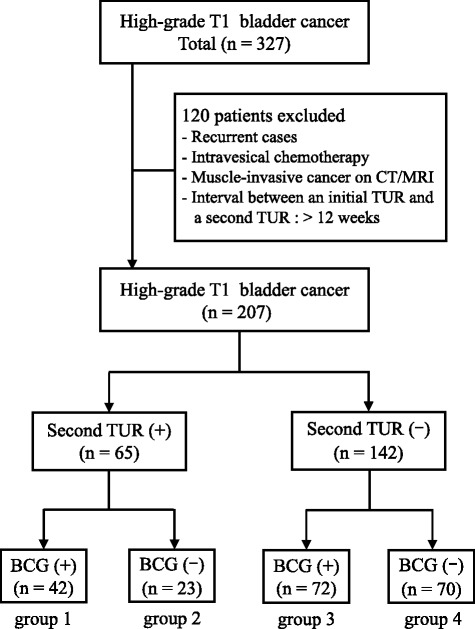
Clinical course of 327 patients diagnosed with high-grade T1 bladder cancer. Group 1: BCG induction instillations after the second TUR, group 2: second TUR alone, group 3: BCG induction instillations alone, group 4: neither a second TUR nor BCG induction instillations. BCG: Bacillus Calmette-Guerin; TUR: transurethral resection

Between April 2008 and September 2013, 327 patients were diagnosed with high-grade T1 bladder cancer at the initial TUR at Nagoya City University Hospital and at five affiliated hospitals. We excluded patients who underwent intravesical chemotherapy after an initial TUR or a second TUR, recurrent cases and patients with clinical muscle-invasive bladder cancer. The clinical course of the 327 patients diagnosed with high-grade T1 bladder cancer is listed in Fig. 1. We also excluded patients with more than 12-week intervals between the initial TUR and the second TUR. Excluding these patients, we retrospectively analyzed 207 patients. Written informed consent was acquired from all participants.

Among consecutive 207 patients, 114 patients received BCG induction instillation (Tokyo 172 strain, purchased from Nihon BCG, Tokyo, Japan) within 2–4 weeks following an initial TUR or a second TUR. Patients younger than 80 years received 80 mg BCG, and patients older than 80 years received 40 mg BCG per instillation (eight times once a week). Some patients terminated BCG induction instillation because of side effects, but a minimum of four applications was performed in all of them. Patients didn’t receive maintenance BCG therapy.

All patients underwent a second TUR with deep muscle fibers in the resected specimen on an initial TUR. In the second TUR, we resected the scar of the initial TUR deeply enough in the muscle fiber and also resected 1 cm around the tumor margin. The initial TUR and the second TUR were performed either by beginners with the instruction of experienced urologists or by experienced urologists themselves. Tumor specimens were classified according to the Union for International Cancer Control–TNM classification 6^th^ edition and the World Health Organization 2004 classification.

During the weekly BCG treatment, a follow-up was performed monitoring patients’ temperature change, bladder irritation, and other symptoms. After termination of the treatment, urine cytology and cystoscopy were performed every 3 months during the first 3 years and every 4–12 months thereafter. We performed enhanced computed tomography in common practice during follow-up, but there was not unified intergroup imaging protocol for the patients with normal urine cytology and no recurrence on cystoscopy. We defined the day of the intravesical recurrence as the day when bladder cancer was pathologically identified.

The end point of this study was the intravesical recurrence. Differences in categorical parameters were assessed using *t*-test, Kruskal–Wallis test, and Fisher’s exact test. The recurrence-free survival (RFS) curves were estimated by the Kaplan–Meier method, and log-rank test was applied to compare survival between groups. To identify risk factors for the intravesical recurrence after the second TUR, we evaluated seven variables (age, gender, urine cytology before the initial TUR, tumor size, tumor number, concomitant carcinoma in situ [CIS] and BCG induction instillation after the initial TUR) by univariate and multivariate analysis using the Cox proportional hazard regression model. A *P*-value < 0.05 was considered to indicate a statistically significant difference. Statistical analyses were performed using the EZR software (Saitama Medical Center, Jichi Medical University, Yakushiji, Japan). This study was approved by our institutional research ethics committee (Nagoya City University ethical board No. 1153).

## Results

The median age was 72.0 years (range: from 39 to 93 years). The patients were followed up until March 2014 and the median follow-up period was 33.5 months (range: from 2.9 to 69.5 months). Patients’ characteristics are shown in Table 1. Of the 207 patients who were diagnosed with high-grade T1 bladder cancer, 65 received a second TUR (31 %) and 142 (69 %) did not (Table 1). BCG induction instillations after the second TUR were performed in 42 patients (group 1) and not in 23 patients (group 2). Of the 142 patients who were treated without a second TUR, 72 patients received BCG induction instillations after the initial TUR (group 3) and 70 patients did not (group 4). Patients in group 4 were older than patients in the other three groups. Patients in group 1 and group 3, who received BCG therapy, had more concomitant CIS and higher EAU recurrence risk score than patients in the two non-BCG treated groups.

**Table 1:** Patients' characteristics in four groups

Characteristics		Group 1 (*n* = 42)		Group 2 (*n* = 23)		Group 3 (*n* = 72)		Group 4 (*n* = 70)	
Follow-up period (months; range)		32.1	(8.9–67.1)	37.1	(8.9–67.1)	32.9	(2.9–64.5)	40.5	(3.7–68.8)
Median age (years; range)		67.8	(39–86)	70.7	(45–84)	72.0	(50–89)	76.6	(43–93)
Gender	Male	35	(83.3 %)	19	(83.3 %)	53	(73.6 %)	53	(75.7 %)
	Female	7	(16.7 %)	4	(16.7 %)	19	(26.4 %)	17	(24.3 %)
Previous history of UTUC	Negative	40	(95.2 %)	23	(100 %)	68	(94.4 %)	64	(91.4 %)
	Positive	2	(4.8 %)	0	(0 %)	4	(5.6 %)	6	(85.7 %)
Urine cytology before the initial TUR	Negative	9	(21.4 %)	5	(21.7 %)	12	(16.7 %)	26	(37.1 %)
	Suspicious positive	23	(54.8 %)	17	(73.9 %)	34	(47.2 %)	31	(44.3 %)
	Positive	9	(21.4 %)	0	(0 %)	20	(27.8 %)	9	(12.9 %)
	Unknown	1	(2.4 %)	1	(43.5 %)	6	(8.3 %)	4	(5.7 %)
Tumor size	<3 cm	29	(69.1 %)	14	(60.9 %)	58	(80.6 %)	13	(18.6 %)
	≥3 cm	13	(31.0 %)	9	(39.1 %)	14	(19.4 %)	57	(81.4 %)
Tumor number	Single	15	(35.7 %)	14	(60.9 %)	17	(23.6 %)	42	(60 %)
	Multiple	27	(64.3 %)	9	(39.1 %)	55	(76.4 %)	28	(40 %)
Concomitant CIS	Negative	37	(88.1 %)	23	(100 %)	60	(83.3 %)	68	(97.1 %)
	Positive	5	(11.9 %)	0	(0 %)	12	(16.7 %)	2	(2.9 %)
EAU recurrence risk	<9	39	(92.9 %)	23	(100 %)	64	(88.9 %)	70	(100 %)
	≥10	3	(7.1 %)	0	(0 %)	8	(11.1 %)	0	(0 %)

**Fig. 2:**
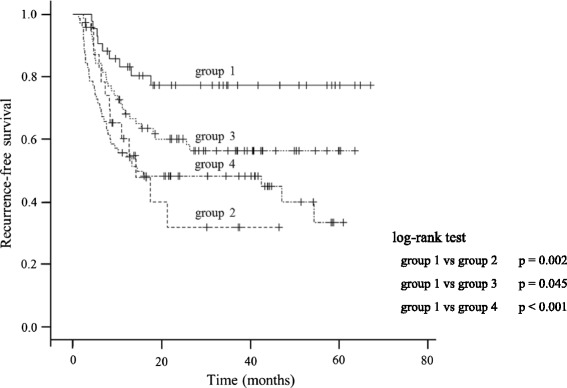
Kaplan–Meier curve of the RFS in the four groups (group 1: BCG induction instillations after the second TUR, group 2: a second TUR alone, group 3: BCG induction instillations alone, group 4: neither a second TUR nor BCG induction instillations). RFS: recurrence-free survival; BCG: Bacillus Calmette-Guerin; TUR: transurethral resection

The median interval between the initial TUR and the second TUR was 6.4 weeks (range: from 1.6 to 11.0 weeks, standard deviation 0.53). Of the 65 patients who underwent a second TUR, residual tumors were detected in 34 patients (52 %). Histopathological findings were pT0 in 31 cases (48 %), dysplasia in 2 cases (3 %), atypical gland in 2 cases (3 %), low-grade urotherial carcinoma in 5 cases (8 %), high-grade urotherial carcinoma pTis/a/1 in 25 cases (38 %). There was no upstaged case. Kaplan–Meier curves of the intravesical RFS of the four groups are shown in Fig. 2. The 1- and 3-year RFS rates of the four groups were 83 %, 77 % (group 1), 60 %, 32 % (group 2), 68 %, 56 % (group 3), and 56 %, 48 % (group 4). Group 1 had longer RFS than the other three groups (group 1 vs group 2, 3, 4, *p* = 0.002, *p* = 0.045, *p* < 0.001, respectively).

Table 2 shows the risk factors for intravesical recurrence after a second TUR determined by univariate and multivariate analyses. BCG induction instillation was the strongest factor to predict intravesical RFS after the second TUR in both univariate and multivariate analyses.

**Table 2:** Risk factors for intravesical recurrence after a second TUR

Variables	Univariate			Multivariate		
	HR (95 % CI)		*p*-value	HR (95 % CI)		*p*-value
Age (≥70)	0.97	(0.85–1.11)	0.68	0.98	(0.79–1.23)	0.87
Gender (female)	1.04	(0.35–3.07)	0.95	1.06	(0.33–3.44)	0.92
Urine cytology before the initial TUR (suspicious positive and positive)	1.52	(0.44–5.22)	0.50	1.77	(0.48–6.54)	0.39
Tumor size (≥3 cm)	1.95	(0.84–4.53)	0.12	2.73	(1.07–7.00)	0.03^*^
Tumor number (multiple)	0.62	(0.27–1.45)	0.27	0.94	(0.37–2.38)	0.89
Concomitant CIS (existence)	1.23	(0.29–5.25)	0.78	3.21	(0.62–16.67)	0.17
BCG induction instillation (without)	0.28	(0.12–0.67)	<0.01^**^	0.20	(0.07–0.57)	<0.01^**^

*p<0.05, **p<0.01 indicate significant differences

Table 3 shows the characteristics and outcome of the recurrent cases in the four groups. Recurrent cases within one year in group 1, 2, 3 and 4 were 7 (17 %), 9 (39 %), 22 (31 %) and 31 (44 %), respectively. With regard to the location of the recurrence in group 1, 2, 3 and 4, recurrence occurred in the same location as the initial TUR in 3 (14 %), 3 (23 %), 20 (69 %) and 29 (41 %) cases, respectively; on the other hand, recurrence occurred in a different location in 7 (33 %), 10 (77 %), 12 (41 %) and 22 (31 %) cases, respectively. On the basis of the RFS in each group and of the location of intravesical recurrence, patients in group 2, who were treated with the second TUR without BCG, had more recurrence at a different site from the initial tumor; patients in group 3, who were treated by BCG induction instillation without a second TUR, had more recurrence at the same site of the initial tumor. These data indicate that either BCG alone or a second TUR alone did not prevent intravesical recurrence.

**Table 3:** Patients' characteristics and outcome of the recurrent cases in the four groups

Characteristics		Group 1 (*n* = 42)		Group 2 (*n* = 23)		Group 3 (*n* = 72)		Group 4 (*n* = 70)	
No. of recurrent cases		9	(21 %)	13	(57 %)	29	(40 %)	39	(56 %)
Recurrence within 1 year		7	(17 %)	9	(39 %)	22	(31 %)	31	(44 %)
Median follow-up period (months; range)		27.8	(11.1–56.5)	20.0	(4.6–62.0)	30.1	(5.6–64.5)	38.2	(3.7–69.5)
Median age (years; range)		74.0	(60–81)	72.9	(45–84)	71.0	(60–84)	78.4	(50–93)
Gender	Male	8	(89 %)	10	(77 %)	20	(69 %)	27	(69 %)
	Female	1	(11 %)	3	(23 %)	9	(31 %)	12	(31 %)
Tumor size^†^	<3 cm	7	(78 %)	5	(38 %)	24	(83 %)	31	(79 %)
	≥3 cm	2	(22 %)	8	(62 %)	5	(17 %)	8	(21 %)
Tumor number^†^	Single	4	(44 %)	8	(62 %)	9	(31 %)	23	(59 %)
	Multiple	5	(56 %)	5	(38 %)	20	(69 %)	16	(41 %)
Concomitant CIS^†^	Negative	7	(78 %)	13	(100 %)	24	(83 %)	38	(97 %)
	Positive	2	(22 %)	0	(0 %)	5	(17 %)	1	(3 %)
EAU recurrence risk^†^	<9	8	(89 %)	13	(100 %)	25	(86 %)	39	(100 %)
	≥10	1	(11 %)	0	(0 %)	4	(14 %)	0	(0 %)
Location of the recurrent tumor^††^	Same	3	(14 %)	3	(23 %)	20	(69 %)	29	(41 %)
	Different	7	(33 %)	10	(77 %)	12	(41 %)	22	(31 %)
	Unknown	0	(0 %)	0	(0 %)	1	(3 %)	2	(3 %)
No. of progressed cases who needed radical cystectomy		3	(7 %)	0	(0 %)	7	(10 %)	4	(6 %)
Outcome	NED	9	(100 %)	12	(92 %)	23	(79 %)	31	(79 %)
	AWD	0	(0 %)	0	(0 %)	4	(14 %)	4	(10 %)
	DOD	0	(0 %)	0	(0 %)	2	(7 %)	2	(5 %)
	DOC	0	(0 %)	1	(8 %)	0	(0 %)	2	(5 %)

† characteristics of the initial TUR, †† compared with the initial TUR

Then, among a total of 31 patients whose pathology of the second TUR was pT0, we administered BCG induction instillation in 12 patients who were treated with a second TUR and in 19 who were not. Only one of the 12 patients (8 %) after BCG induction instillation following the second TUR had recurrence, and the location of the recurrent tumor was the same as the initial tumor. On the other hand, 11 of 19 patients (58 %) after the second TUR without BCG had recurrence (Table 4). Ten of these 11 cases had recurrence at a different site from the initial tumor; only one had recurrence at the same site of the initial tumor. These results indicate that high-grade T1 bladder cancer can recur even after the tumor has been completely resected, and that BCG induction instillation can prevent ectopic intravesical recurrence after complete resection.

**Table 4:** Characteristics and outcome of patients whose pathology of the second TUR was pT0

Characteristics		BCG(+) (*n* = 12)		BCG(−) (*n* = 19)		*p*-value
No. of recurrent cases		1	(8 %)	11	(58 %)	
Recurrence within 1 year		1	(8 %)	8	(42 %)	
Median follow-up period (months; range)		17.8	(5.6–33.9)	11.4	(4.1–37.6)	
Median age (years; range)		65.5	(39–86)	71.9	(45–84)	0.22
Gender	Male	8	(67 %)	15	(79 %)	0.89
	Female	4	(33 %)	4	(21 %)	
Tumor size	<3 cm	8	(67 %)	11	(58 %)	0.30
	≥3 cm	4	(33 %)	8	(42 %)	
Tumor number	Single	6	(50 %)	11	(58 %)	0.63
	Multiple	6	(50 %)	8	(42 %)	
Concomitant CIS	Negative	8	(67 %)	19	(100 %)	0.19
	Positive	4	(33 %)	0	(0 %)	
EAU recurrence risk	<9	11	(92 %)	19	(100 %)	0.07
	≥10	1	(8 %)	0	(0 %)	
Location of the recurrent tumor^†^	Same	1	(8 %)	1	(5 %)	
	Different	0	(0 %)	10	(53 %)	

† compared with the initial TUR

## Discussion

A second TUR plays an important role in both resecting a residual tumor and detecting staging error. Although recent studies on second TUR referred to both applications [2–6], we mainly focused on the resection of the residual tumor in order to clarify the effect of BCG induction instillation following a second TUR. Most studies on second TURs analyzed the efficacy of a second TUR regardless of BCG induction instillation [2–6, 8]. However, in the present study, we found that BCG induction instillation should be performed after the second TUR.

The 1- and 3- year RFS rates after the second TUR without BCG induction instillations have been described to be 82 % and 65–68 %, respectively [2, 3]. In the present study, the 1- and 3- year RFS rates were 68 % and 56 %, respectively, after BCG induction instillations following an initial TUR; 60 % and 32 %, respectively, after the second TUR without BCG; and 84 % and 78 %, respectively, after the second TUR followed by BCG instillations. Compared with the previous reports, our data showed lower RFS in the group without BCG following the second TUR, whereas higher RFS in the group with BCG following the second TUR.

The recurrence of bladder cancer after the initial TUR is either represented by the residual tumor due to incomplete resection, or by small lesions that have been overlooked, or by new occurrence caused by implantation of the circulating tumor cells [9]. Although BCG is thought to be effective against recurrence, BCG therapy against residual tumors has received little attention in the literatures. Herr HW reported that 67 % of T1 bladder cancers after BCG therapy without a second TUR failed to respond to BCG, whereas only 24 % of T1 bladder cancers after BCG induction instillations following second TUR failed to respond to BCG [4]. He found that BCG had anti-tumor effect, particularly against CIS, but not against residual tumors.

According to recent reports, the rates of residual tumors after an initial TUR were 33–75 % [1, 3, 5, 6]. In our study we found residual tumors in 54 % of the cases. Our study, characterized by a relatively high rate of residual tumors at the initial TUR and early high recurrence rate at the same sites of the initial TUR, indicates that BCG induction instillations are not effective against residual tumors. Comparison of RFS between group 1 and group 3 indicates that a second TUR could prevent recurrence at the same sites of the initial TUR and that BCG induction instillation was not effective against the residual tumors. Moreover, among the patients whose pathology of the second TUR was pT0, only 8 % who received BCG induction instillations showed recurrence. On the other hand, in 55 % of the patients who didn’t receive BCG following the second TUR showed recurrence. Therefore, taking into account the high recurrence rates of the patients treated by a second TUR alone with a pT0 pathology, it is recommended to administer BCG therapy following the second TUR, in order to prevent ectopic intravesical recurrence.

The treatment of all second TURs with BCG induction instillations might be argued. In our study, we had to treat all the recurrent cases after second TUR without BCG with another TUR. Although in this study we didn’t analyze the effect of BCG following a second TUR on overall survival, BCG following a second TUR might have the same effect as BCG following an initial TUR [10]. In addition, BCG often develops many side effects, especially in elderly people or patients who take antiplatelet or anticoagulant agents [11–13]. Recently, preclinical studies using a novel engineered mycobacterium vaccine have been conducted to overcome the limitations of BCG therapy [14]. Therefore, for preventing recurrence, in the future it may be feasible to add this new immunotherapy induction instillations following a second TUR.

Our study has some limitations. There were no exact criteria for administering BCG or for undergoing a second TUR. The treatment after the initial TUR was decided according to the doctors’ choice or to the policy of each hospital. Moreover, a further large cohort study is needed.

In conclusion, our study indicates that BCG induction instillation have limited efficacy against unresected tumors and that BCG induction instillation following a second TUR clearly prevent intravesical recurrence, even if there is no residual tumor on the second TUR. Further prospective randomized investigations are necessary to understand the role of BCG induction instillation following a second TUR.

## Conclusions

BCG induction instillations following a second TUR clearly prevent intravesical recurrence, even if there is no residual tumor on the second TUR.

## Abbreviation

TUR: Transurethral resection
BCG: Bacillus Calmette-Guerin
RFS: Recurrence-free survival
CIS: Carcinoma in situ
EAU: European Association of Urologists
